# Genetic variations in *TERC* and *TERT* genes are associated with lung cancer risk in a Chinese Han population

**DOI:** 10.18632/oncotarget.22329

**Published:** 2017-11-06

**Authors:** Gang Ye, Nan Tan, Chenyang Meng, Jingjie Li, Li Jing, Mengdan Yan, Tianbo Jin, Fulin Chen

**Affiliations:** ^1^ Key Laboratory of Resource Biology and Biotechnology in Western China, Ministry of Education, School of Life Sciences, Northwest University, Xi’an, Shaanxi 710069, China; ^2^ First Affiliated Hospital, School of Medicine, Shihezi University, Shihezi, Xinjiang 832008, P. R. China; ^3^ Department of Cadre's Ward, Xi’an No.1 Hospital, Xi’an, Shaanxi 710002, China; ^4^ Graduate School of Inner Mongolia Medical University, Hohhot, Inner Mongolia 010050, China; ^5^ Xi’an Tianqin Precision Medical Institute, Xi’an, Shaanxi 710075, China

**Keywords:** single nucleotide polymorphism (SNP), TERC, TERT, lung cancer

## Abstract

The study was aimed to explore whether the *TERT* and *TERC* polymorphisms are associated with the lung cancer risk. Five *TERC* and *TERT* polymorphisms were genotyped from 554 lung cancer patients and 603 healthy controls. We used χ^2^ test, genetic model, linkage disequilibrium (LD) and haplotype analyses to evaluate the association between the polymorphisms and lung cancer risk. We found that the allele “C” of rs10936599 (*TERC*) and the allele “T” of rs10069690 (*TERT*) were associated with increased risk of lung cancer (OR = 1.32, 95% CI: 1.12-1.55, *P* = 0.001; OR = 1.41, 95% CI: 1.14-1.76, *P* = 0.002, respectively). The genotype of “CC” of rs10936599, genotype “CT” of rs10069690 and genotype “GG and “AG” of rs2853677 were also associated with increased the risk of lung cancer. In addition, rs10936599 under the dominant, recessive and log-additive models; rs10069690 under the dominant, overdominant and log-additive models; rs2853677 under the dominant and log-additive models were found to be associated with increased lung cancer risk. The SNP rs2242652 was found to be associated with an increased lung cancer risk under the dominant and overdominant models without adjustment. The haplotype “TA” of *TERT* was also associated with an increased risk of lung cancer. Our study indicated that rs10936599 (*TERC*) and rs10069690, rs2242652 and rs2853677 in *TERT* and haplotype “TA” of *TERT* were revealed as risk factors of lung cancer in a Chinese Han population. However, it required to verify our result and investigate the function genetic variants and mechanism of lung cancer.

## INTRODUCTION

Lung cancer is a malignant tumor with high morbidity and mortality. Lung cancer as the leading cause of cancer-related deaths caused occurred in 1.8 million people and resulted in 1.6 million deaths worldwide in 2012 [1]. It was reported that lung cancer is the highest incidence of malignant tumors in China with the annual new cases about 705,000 [2]. The cigarette smoking, environmental pollution, radon exposure, occupational exposure, and pre-existing lung disease were found to be important contributors to lung cancer risk [3, 4]. However, not all people with these risk factors develop lung cancer. About 8% of lung cancer is due to inherited factors [5]. Previous studies have identified multiple susceptibility polymorphisms in some genes that are associated with the risk of lung cancer, such as *TERT* [6, 7], *TP63* [8] *CLPTM1L* [9, 10], *PSMA4* [11], *CHRNA3* [12], *CHRNA5* [13], *CRP* and *GPC5* [14], and so on.

Telomeres are special functional structure at the end of chromosomes and they have been deemed as a crucial role in maintaining the stability and integrity of the genome [15]. Telomere dysfunction could lead to chromosomal instability and the increase of carcinoma susceptibility so that the cells would get growth advantages, even develop into tumor cells [16, 17]. The *TERT* (telomerase reverse transcriptase) gene, encoding the catalytic subunit of the telomerase, is primarily regulated at the level of transcriptional initiation. The *TERC* (telomerase RNA component) gene, encoding the subunit of the enzyme, provides the template for the synthesis of the TTAGGG repeats. The two major telomerase genes are maintained telomere DNA length, chromosomal stability, and cellular immortality.

A number of studies have reported that single nucleotide polymorphisms (SNPs) of *TERT* and *TERC* gene are associated with a significantly higher susceptibility to multiple types of cancers [6, 17–20]. But few studies were reported on the association between SNPs in *TERC* gene and the risk of lung cancer. In the study, we conducted a case-control study consisting of 554 lung cancer patients and 603 healthy controls to investigate whether SNPs in *TERT* and *TERC* are associated with lung cancer risk in the Chinese Han population, and further to verify whether the conclusion we drew was in accord with the former studies.

## RESULTS

In our study, the basic characteristics were shown in the Table 1. There were 416 males and 138 females in the case group with a mean age of 58.18±10.534, 496 males and 134 females in the control group with a mean age of 48.24±13.053. There was a statistical difference in age between two groups and no significant difference in gender. We adjusted gender and age in later multivariate unconditional logistic regression analysis in order to eliminate those residual confounding effects.

**Table 1:** Basic characteristics of the cases and controls

Characteristic		Case (n=554)	Control (n=603)	*P*-value
Gender	Male	416	469	1.16^a^
	Female	138	134	
Age (year; mean ± SD)		58.18±10.534	48.24±13.053	**<0.001**^b^

SD: Standard deviation

^a^*P* value was calculated by Welch's t test;

^b^*P* value was calculated by Pearson's χ ^2^ test.

The allele distributions and minor allele frequency (MAF) of the SNPs and the results of Hardy-Weinberg equilibrium (HWE) test were shown in the Table 2. Genotype distributions of the five SNPs appeared compatible with HWE in control subjects (all HWE*-P* > 0.05). In the allele model, the allele “C” of rs10936599 (*TERC*) and the allele “T” of rs10069690 (*TERT*) were found to be associated with increased the risk of lung cancer (OR = 1.32, 95% CI: 1.12-1.55, *P* = 0.001; OR = 1.41, 95% CI: 1.14-1.76, *P* = 0.002, respectively). At the level of *P*<0.002 (0.05/(5^*^5)), rs10936599 in the *TERC* remained significant after Bonferroni correction.

**Table 2:** Association between the SNPs of *TERC* and *TERT* and the risk of lung cancer

SNP ID	Position	Band	Alleles A/B	Gene(s)	MAF		HWE-*P*	OR (95 % CI)	*P*
					Case	Control			
rs10936599	169492101	3q26.2	C/T	*TERC*	0.493	0.425	0.6169	1.32 (1.12-1.55)	**0.001**
rs10069690	1279790	5p15.33	T/C	*TERT*	0.196	0.147	0.1899	1.41 (1.14-1.76)	**0.002**
rs2242652	1280028	5p15.33	A/G	*TERT*	0.190	0.163	0.5489	1.21 (0.98-1.50)	0.079
rs2853677	1287194	5p15.33	G/A	*TERT*	0.403	0.366	0.7929	1.17 (1.00-1.39)	0.060
rs2853676	1288547	5p15.33	T/C	*TERT*	0.175	0.154	0.7565	1.16 (0.93-1.45)	0.184

SNP: single nucleotide polymorphism; MAF: Minor allele frequency; HWE: Hardy–Weinberg equilibrium; OR: Odds ratio; 95 % CI: 95 % Confidence interval

*P* values were calculated using two-sided Chi-squared test.

*P* < 0.05 indicates statistical significance.

We further assessed the association between the five SNPs and lung cancer risk under five genetic models including codominant, dominant, recessive, overdominant model and log-additive models (Table 3). In the codominant model, the genotype of “CC” of rs10936599 was associated with increased lung cancer risk compared to the genotype “TT” (OR = 1.72, 95% CI: 1.24-2.38, *P =* 0.004; adjusted OR = 1.72, 95% CI: 1.21-2.45, *P =* 0.010, respectively). In addition, rs10936599 was found to be associated with increased lung cancer risk under the dominant model (OR = 1.35, 95% CI: 1.05-1.73, *P =* 0.020; adjusted OR = 1.36, 95% CI: 1.04-1.79, *P =* 0.026), recessive model (OR = 1.53, 95% CI: 1.16-2.03, *P =* 0.003; adjusted OR = 1.52, 95% CI: 1.12-2.06, *P =* 0.007) and log-additive model (OR = 1.30, 95% CI: 1.11-1.53, *P =* 0.001; adjusted OR = 1.30, 95% CI: 1.09-1.55, *P =* 0.003).

**Table 3:** Genetic model analyses of the association between the SNPs and lung cancer risk

SNP-ID	Model	Genotype	Case (%)	Control (%)	Without adjustment		With adjustment	
					OR (95% CI)	*P*	OR (95% CI)	*P*
rs10936599	Codominant	T/T	151 (27.4%)	203 (33.7%)	1	**0.004**	1	**0.010**
		C/T	258 (46.7%)	288 (47.8%)	1.20 (0.92-1.58)		1.22 (0.91-1.64)	
		C/C	143 (25.9%)	112 (18.6%)	1.72 (1.24-2.38)		1.72 (1.21-2.45)	
	Dominant	T/T	151 (27.4%)	203 (33.7%)	1	**0.020**	1	**0.026**
		C/T-C/C	401 (72.6%)	400 (66.3%)	1.35 (1.05-1.73)		1.36 (1.04-1.79)	
	Recessive	T/T-C/T	409 (74.1%)	491 (81.4%)	1	**0.003**	1	**0.007**
		C/C	143 (25.9%)	112 (18.6%)	1.53 (1.16-2.03)		1.52 (1.12-2.06)	
	Overdominant	T/T-C/C	294 (53.3%)	315 (52.2%)	1	0.730	1	0.850
		C/T	258 (46.7%)	288 (47.8%)	0.96 (0.76-1.21)		0.98 (0.76-1.25)	
	Log-additive	---	---	---	1.30 (1.11-1.53)	**0.001**	1.30 (1.09-1.55)	**0.003**
rs10069690	Codominant	C/C	354 (64%)	436 (73.4%)	1	**0.002**	1	**0.003**
		C/T	181 (32.7%)	141 (23.7%)	1.58 (1.22-2.05)		1.62 (1.22-2.15)	
		T/T	18 (3.2%)	17 (2.9%)	1.30 (0.66-2.57)		1.38 (0.66-2.88)	
	Dominant	C/C	354 (64%)	436 (73.4%)	1	**0.001**	1	**0.001**
		C/T-T/T	199 (36%)	158 (26.6%)	1.55 (1.21-1.99)		1.59 (1.21-2.09)	
	Recessive	C/C-C/T	535 (96.8%)	577 (97.1%)	1	0.700	1	0.630
		T/T	18 (3.2%)	17 (2.9%)	1.14 (0.58-2.24)		1.20 (0.58-2.50)	
	Overdominant	C/C-T/T	372 (67.3%)	453 (76.3%)	1	**0.001**	1	**0.001**
		C/T	181 (32.7%)	141 (23.7%)	1.56 (1.21-2.03)		1.59 (1.20-2.11)	
	Log-additive	---	---	---	1.41 (1.13-1.76)	**0.002**	1.45 (1.14-1.83)	**0.002**
rs2242652	Codominant	G/G	358 (64.9%)	425 (70.5%)	1	0.100	1	0.150
		G/A	178 (32.2%)	160 (26.5%)	1.32 (1.02-1.71)		1.32 (1.00-1.74)	
		A/A	16 (2.9%)	18 (3%)	1.06 (0.53-2.10)		1.06 (0.50-2.23)	
	Dominant	G/G	358 (64.9%)	425 (70.5%)	1	**0.041**	1	0.061
		G/A-A/A	194 (35.1%)	178 (29.5%)	1.29 (1.01-1.66)		1.29 (0.99-1.69)	
	Recessive	G/G-G/A	536 (97.1%)	585 (97%)	1	0.930	1	0.940
		A/A	16 (2.9%)	18 (3%)	0.97 (0.49-1.92)		0.97 (0.46-2.04)	
	Overdominant	G/G-A/A	374 (67.8%)	443 (73.5%)	1	**0.033**	1	0.051
		G/A	178 (32.2%)	160 (26.5%)	1.32 (1.02-1.70)		1.32 (1.00-1.73)	
	Log-additive	---	---	---	1.21 (0.98-1.51)	0.079	1.21 (0.96-1.53)	0.110
rs2853677	Codominant	A/A	191 (34.5%)	244 (40.5%)	1	0.110	1	**0.031**
		A/G	279 (50.4%)	277 (45.9%)	1.29 (1.00-1.66)		1.32 (1.00-1.73)	
		G/G	84 (15.2%)	82 (13.6%)	1.31 (0.91-1.87)		1.60 (1.08-2.37)	
	Dominant	A/A	191 (34.5%)	244 (40.5%)	1	0.036	1	**0.015**
		A/G-G/G	363 (65.5%)	359 (59.5%)	1.29 (1.02-1.64)		1.38 (1.06-1.79)	
	Recessive	A/A-A/G	470 (84.8%)	521 (86.4%)	1	0.450	1	0.082
		G/G	84 (15.2%)	82 (13.6%)	1.14 (0.82-1.58)		1.37 (0.96-1.97)	
	Overdominant	A/A-G/G	275 (49.6%)	326 (54.1%)	1	0.130	1	0.250
		A/G	279 (50.4%)	277 (45.9%)	1.19 (0.95-1.50)		1.16 (0.90-1.49)	
	Log-additive	---	---	---	1.18 (0.99-1.39)	0.060	1.28 (1.06-1.54)	**0.009**
rs2853676	Codominant	C/C	380 (68.6%)	429 (71.3%)	1	0.270	1	0.150
		C/T	154 (27.8%)	160 (26.6%)	1.09 (0.84-1.41)		1.14 (0.86-1.52)	
		T/T	20 (3.6%)	13 (2.2%)	1.74 (0.85-3.54)		2.01 (0.93-4.31)	
	Dominant	C/C	380 (68.6%)	429 (71.3%)	1	0.320	1	0.180
		C/T-T/T	174 (31.4%)	173 (28.7%)	1.14 (0.88-1.46)		1.21 (0.92-1.59)	
	Recessive	C/C-C/T	534 (96.4%)	589 (97.8%)	1	0.140	1	0.085
		T/T	20 (3.6%)	13 (2.2%)	1.70 (0.84-3.44)		1.93 (0.90-4.13)	
	Overdominant	C/C-T/T	400 (72.2%)	442 (73.4%)	1	0.640	1	0.460
		C/T	154 (27.8%)	160 (26.6%)	1.06 (0.82-1.38)		1.11 (0.84-1.47)	
	Log-additive	---	---	---	1.16 (0.93-1.44)	0.180	1.23 (0.97-1.56)	0.085

SNP: single nucleotide polymorphism; OR: Odds ratio; 95% CI: 95% Confidence interval

*P* < 0.05 indicates statistical significance.

In the codominant model, the genotype “CT” of rs10069690 was also associated with increased lung cancer risk compared to the genotype “CC” (OR = 1.58, 95% CI: 1.22-2.05, *P =* 0.002; adjusted OR = 1.62, 95% CI: 1.22-2.15, *P =* 0.003). We also found that rs10069690 was associated with an increased the risk of lung cancer under the dominant model (OR = 1.55, 95% CI: 1.21-1.99, *P =* 0.001; adjusted OR = 1.59, 95% CI: 1.21-2.09, *P =* 0.001), overdominant model (OR = 1.56, 95% CI: 1.21-2.03, *P =* 0.001; adjusted OR = 1.59, 95% CI: 1.20-2.11, *P =* 0.001), and log-additive model (OR = 1.41, 95% CI: 1.13-1.76, *P =* 0.002; adjusted OR = 1.45, 95% CI: 1.14-1.83, *P =* 0.002).

In the codominant model, the genotype “GG and “AG” of rs2853677 were associated with increased lung cancer risk compared to the genotype “AA” (adjusted OR = 1.32, 95% CI: 1.00-1.73, *P =* 0.031; adjusted OR = 1.60, 95% CI: 1.08-2.37, *P =* 0.031) after adjustment for gender and age. The SNP rs2853677 was also associated with increased the risk of lung cancer under dominant model (adjusted OR = 1.38, 95% CI: 1.06-1.79, *P =* 0.015) and log-additive model (adjusted OR = 1.28, 95% CI: 1.06-1.54, *P =* 0.009) after adjustment for gender and age.

However, the SNP rs2242652 was found to be associated with an increased risk of lung cancer under the dominant model (OR = 1.29, 95% CI: 1.01-1.66, *P =* 0.041) and overdominant model (OR = 1.32, 95% CI: 1.02-1.70, *P =* 0.033) without adjustment. At the level of *P*<0.002 (0.05/(5^*^5)), only the SNP rs10069690 in the *TERT* remained significant after Bonferroni correction.

**Figure 1:**
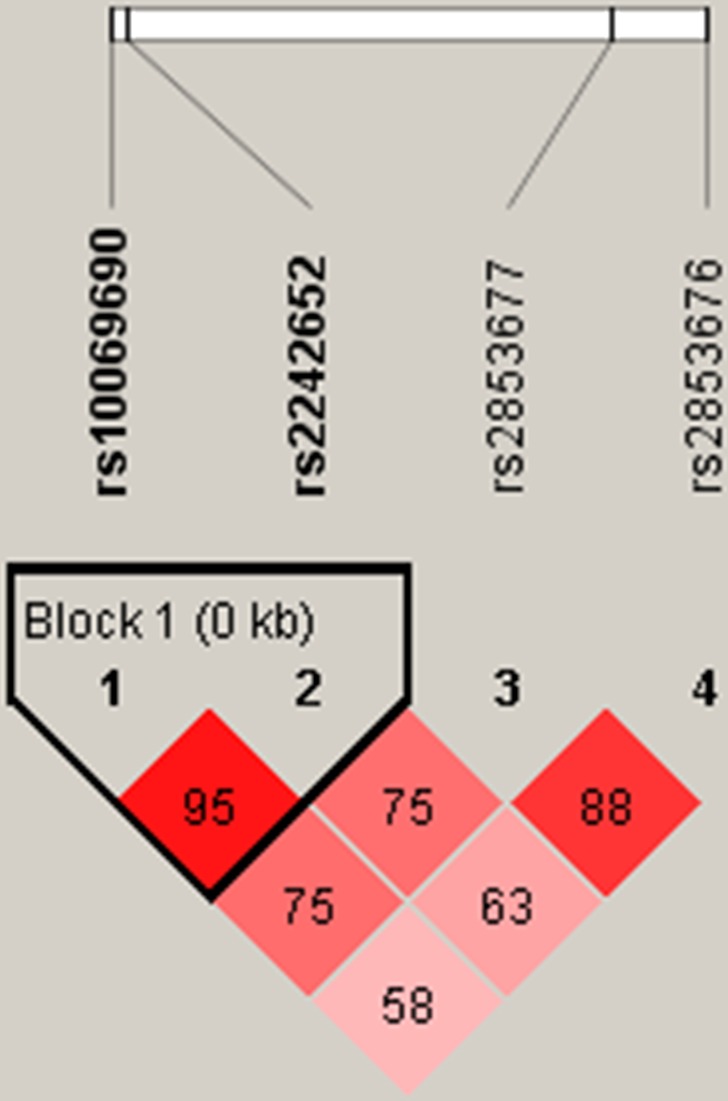
Haplotype block map for SNPs of the TERT gene LD is displayed by standard color schemes with bright red for very strong LD (LOD > 2, D’ = 1), pink red (LOD > 2, D’< 1) and blue (LOD < 2, D’= 1) for partial linkage, and white (LOD < 2, D’< 1) for complete recombination.

Finally, there was a strong linkage between the candidate SNPs in the *TERT* gene (rs10069690 and rs2242652), as shown in the Figure 1. Furthermore, we found that the haplotype “TA” of *TERT* was associated with a 1.32-fold increased the risk of lung cancer (OR = 1.32, 95% CI: 1.06 - 1.65, *P =* 0.019) (Table 4).

**Table 4:** Haplotype frequency and their association with lung cancer risk

SNPs	Haplotype	Freq%		*P*^a^	OR (95%CI)	*P*^b^
		case	control			
rs10069690|rs2242652	CG	0.800	0.832	-	1	-
	TA	0.187	0.146	**0.014**	1.32 (1.06 - 1.65)	**0.019**

OR: Odds ratio; 95% CI: 95% Confidence interval

^a^*P* value was calculated by logistic regression crude analysis.

^b^*P* value was calculated by logistic regression analysis adjusted by gender and age.

*P* < 0.05 indicates statistical significance.

## DISCUSSION

In the case-control study, we investigated the association between SNPs in *TERC*, *TERT* genes and the risk of lung cancer in Chinese Han population. The results showed that rs10936599 in *TERC* and rs10069690, rs2242652 and rs2853677 in *TERT* were revealed as risk factors of lung cancer. Besides, the haplotype “TA” of *TERT* gene was also associated with an increased risk of lung cancer.

Telomeres at the ends of chromosomes play a significant role in maintaining the stability and integrity of the genome [15]. During somatic cell divisions, telomeres progressively shorten. While the telomeres turn into a critical threshold, they may lead to cell senescence or apoptosis [21]. Previous studies reported that telomere length was associated with lung cancer risks, but the conclusions were inconsistent [22–24]. *TERT* gene at 5p15.33 encodes the catalytic subunit of telomerase reverse transcriptase, which is an important component of telomerase complex and associated with telomere length. *TERC* gene located at human chromosome 3q26.2 region, which encodes the RNA component of human telomerase. TERC serving as a template for telomere elongation can affect telomere homeostasis.

Recent studies indicated that rs10936599 in *TERC* was associated with telomere length and with an increased risk of cancers, but the conclusions were inconsistent [20, 25, 26]. To date, there no report on the association between rs10936599 and lung cancer risk. In our study, rs10936599 was found to be associated an increased risk of lung cancer. We suggested that rs10936599 has association with lung cancer risk may by influencing the balancing the telomere length. Further studies need to verify the assumption. Gao et al. revealed that rs10069690 in *TERT* was associated with an increased risk of lung cancer under dominant model [6]. In our study, there was a strong correlation between rs10069690 and lung cancer risk. Therefore, more powerful evidence in our study was provided to prove rs10069690 act as an increased risk of lung cancer. It has been reported that the rs2242652 allele of *TERT* influences telomere length, which was associated with risk of several cancers [27–29]. A study found that rs2242652 was associated with an increased lung cancer risk under the dominant model in the Han Chinese population [6]. However, our results showed that rs2242652 increased the risk of lung cancer in dominant model without adjustment. Therefore, it needs to be affirmed by further large-scale samples studies.

Previous studies indicated that the allele of rs2853677 in *TERT*, disrupted the Snail1 binding site, causing derepression of *TERT* transcription in response to Snail1 upregulation, was associated with a high risk of lung adenocarcinoma [17, 30, 31]. We found rs2853677 was also associated with an increased lung cancer risk. A meta-analysis study showed that rs2853676 (*TERT*) was associated with an increased risk of lung adenocarcinoma in Caucasians population [32]. However, in our study, no association was found between rs2853676 and lung cancer risk in the Chinese Han population. The difference in sample size and races may be the result of inconsistencies in the findings.

Finally, there are some potential limitations. First, smoking and other risk factors was not included due to lack of corresponding clinical information. Second, the associations between SNPs and telomere length, telomere length and lung cancer risk were not performed in our study. Moreover, the biological function of each SNP was not explored and these deserved further study.

In conclusion, the study demonstrated that rs10936599 (*TERC*), rs10069690, rs2242652 and rs2853677 in *TERT*, and haplotype “TA” of *TERT* are associated with increased risk of lung cancer in Chinese Han population. Our study not only indicated a new SNP (rs10936599) in *TERC* which as a risk factor of lung cancer, but supplemented and verified the findings reported before, thus there could be more powerful evidences and they can be used as diagnostic and prognostic markers in clinical studies of lung cancer patients.

## MATERIALS AND METHODS

### Study participants

A total of 554 patients with lung cancer and 603 healthy controls were recruited in our study. All the patients were treated in the First Affiliated Hospital of Xi’an Jiaotong University between January 2014 and August 2016. All demographic and related clinical data including age, gender were collected through a review of medical records. Inclusion criteria included patients who recently diagnosed with primary lung cancer (confirmed by histopathological examination) and patients who had not yet received any chemotherapy or radiotherapy. Patients with other types of cancers were excluded. The healthy controls were randomly recruited from unrelated subjects living Xi’an and nearby. Moreover, people with chronic disease involving brain, liver, and heart were excluded from our study.

We obtained the informed consent from all of the participants and the study protocols were approved by the Ethics Committee of First Affiliated Hospital of Xi’an Jiaotong University and in accordance with the ethical guidelines of the Declaration of Helsinki.

### SNP selection and genotyping

We collected 5mL peripheral blood venous in an EDTA tube from each subject. Genomic DNA was extracted from whole blood samples using the Gold Mag-Mini Whole Blood Genomic DNA Purification Kit according to the manufacturers’ instructions (GoldMag. Co. Ltd., Xi’an, China). The DNA concentration and purity were measured by NanoDrop 2000 spectrophotometer (Thermo Fisher Scientific, Waltham, MA, USA). We reviewed the literatures related to association between *TERT* and *TERC* polymorphisms and lung cancer and other cancers then selected five SNPs in *TERC* (rs10936599) and *TERT* (rs10069690, rs2242652, rs2853677 and rs2853676) with the minor allele frequencies (MAF) ≥5% in the Chinese Han Beijing (CHB) population by using HapMap database [6, 31–33]. The sequences of primers for amplification and extension were designed by the Sequenom MassARRAY Assay Design 3.0 software (Sequenom, Inc, San Diego, CA, USA), as shown in Table 5. Genotyping was performed using a Sequenom MassARRAY platform (Sequenom, San Diego, CA, USA) in accordance with the manufacturer's protocol. Sequenom Typer 4.0 software was used to perform data management and analyses.

**Table 5:** The sequences of primers for SNPs

SNP-ID	2^nd^-PCRP	1^st^-PCRP	UEP-SEQ
rs10936599	ACGTTGGATGCAAGGGTAAAATTCCATTCTG	ACGTTGGATGTTCCCGCTGTTTGTTCAGTC	ATGCAGTATTCGCACCA
rs10069690	ACGTTGGATGATGTGTGTTGCACACGGGAT	ACGTTGGATGCCTGTGGCTGCGGTGGCTG	GGGATCCTCATGCCA
rs2242652	ACGTTGGATGAGGCTCTGAGGACCACAAGA	ACGTTGGATGACAGCAGGACACGGATCCAG	gtcgGAGGACCACAAGAAGCAGC
rs2853677	ACGTTGGATGGCAAGTGGAGAATCAGAGTG	ACGTTGGATGATCCAGTCTGACAGTCGTTG	gggtAATCAGAGTGCACCAG
rs2853676	ACGTTGGATGCAAAACTAAGACCCAAGAGG	ACGTTGGATGTGTCTCCTGCTCTGAGACC	agatGGAAGTCTGACGAAGGC

SNP: Single nucleotide polymorphism; PCRP: Polymerase chain reaction primer; UEP: unique base extension primer; SEQ: Single base extension reaction

Sequences are written in the 5’→3’ (left to right) orientation.

### Statistical analysis

The SPSS version 20.0 statistical package (SPSS, Chicago, IL, USA) and Microsoft Excel were used for all statistical analyses. We performed Chi-squared/ exact test to compare the expected frequencies of the genotypes in the controls in order to assess the Hardy-Weinberg equilibrium (HWE) of each SNP. All of the minor alleles were deemed as risk alleles for lung cancer susceptibility. The differences in frequency distributions of alleles were compared between cases and controls by Chi-squared test. We performed five genetic models analyses (codominant, dominant, recessive, overdominant and log-additive) to evaluate associations between the SNPs and risk of lung cancer. The odds ratios (ORs) and 95% confidence intervals (CIs) were used for crude logistic regression analysis and logistic regression analysis adjusted by gender and age. We used the Haploview software package (version 4.2) to analyze the linkage disequilibrium (LD) and haplotypes. For LD analysis and haplotype construction, genotype data of control groups were used to estimate the degree of LD by measures D’, and the D’ value > 0.8 demonstrated the related SNPs formed one haplotype block. The *P-*value < 0.05 was considered statistically significant. Besides, The *P-*value < 0.002(0.05/(5^*^5)) indicates statistical significance after Bonferroni correction. All statistical tests were two-sided.
